# Risk factors for ninety-day readmission following cervical surgery: a meta-analysis

**DOI:** 10.1186/s13018-022-03377-x

**Published:** 2022-11-03

**Authors:** Dongping Wang, Wenqing Liao, Haoshi Hu, Xiaoling Lei, Xinze Zheng, Daxiang Jin

**Affiliations:** 1 Department of Orthopedics, The First Affiliated Hospital of Guangzhou University of Chinese Medicine, Guangzhou, 510405 Guangdong China; 2 Department of Physical Medicine and Rehabilitation, Hubei Provincial Hospital of Integrated Chinese and Western Medicine, Wuhan, 430015 Hubei China

**Keywords:** Risk factors, Ninety‐day readmission, Cervical surgery, Meta-analysis

## Abstract

**Background:**

As an important evaluation index after cervical surgery, ninety-day readmission is gradually being valued. Our study collected the latest published relevant studies, analyzed the risk factors of ninety-day readmission after cervical surgery, and continuously improved the postoperative rehabilitation plan. This study focuses on two research hotspots: (1) What is the rate of ninety-day readmission after cervical surgery? (2) What are the risk factors affecting the ninety-day readmission?

**Methods:**

Based on the Cochrane Library, PubMed, Web of Science, and Embase databases, this study searched for studies about ninety-day readmission after cervical surgery, from the establishment of the database to August 1, 2022. The evaluation indicators are as follows: age, American Society of Anesthesiology physical status (ASA) class, diabetes, hypertension, chronic heart diseases, chronic lung diseases, income, and payments for hospitalization. The meta-analysis was performed using Review Manager 5.4.

**Results:**

Seven studies with 222,490 participants were eligible for our meta-analysis. The analysis displayed that there were statistically significant differences in the age (MD = − 4.60, 95%CI − 4.89–4.31, *p* < 0.001), diabetes (OR = 0.60, 95%CI 0.56–0.64, *p* < 0.00001), hypertension (OR = 0.40, 95%CI 0.30–0.54, *p* < 0.00001), chronic heart diseases (OR = 0.05, 95%CI 0.01–0.19, *p* < 0.00001), chronic lung diseases (OR = 0.46, 95%CI 0.43–0.49, *p* < 0.00001), income (OR = 2.85, 95%CI 1.82–4.46, *p* < 0.00001), and payments for hospitalization (OR = 2.29, 95%CI 1.14–4.59, *p* = 0.02) between readmission and no readmission groups. In terms of the ASA, there was no difference on the ninety-day readmission (*p* = 0.78).

**Conclusion:**

Age, diabetes, hypertension, chronic heart diseases, chronic lung diseases, income, and payments for hospitalization are the risk factors of ninety‐day readmission following cervical surgery.

## Introduction

With the deepening of the concept of minimally invasive spine surgery, the hospital stay after cervical surgery is reduced [1]. However, the shortening of hospital stay may result in a lack of observation of the patient’s postoperative rehabilitation progress [2]. Some complications of cervical surgery are covered up by treatments such as drugs and home physical rehabilitation. The potential risk of unplanned rehospitalization for postoperative patients increases [3, 4]. The ninety-day readmission is an important indicator in the medical quality evaluation system. It is an important reason for the waste of medical resources and the aggravation of the economic burden on the patient’s family. The American health system spends more than $17 billion annually on unplanned readmissions [5]. Therefore, controlling the postoperative readmission rate is undoubtedly an important part of saving medical operating costs.

In 1959, Boucher completed the world’s first case of spinal pedicle screw arthrodesis [6]. The technology of spinal internal fixation began to develop rapidly, especially in the field of cervical spine surgery. In the face of complex cervical degeneration, trauma, tumors and infections, new cervical surgery techniques have given patients more treatment options [7]. The number of cervical surgeries has also increased. Compared to other parts of the operation, cervical surgery has the characteristics of larger trauma, higher risk and higher cost [8, 9]. Once unplanned readmission occurs, it will bring heavy mental burden and economic pressure to the patients and at the same time increase the difficulty of diagnosis and treatment for physicians. Ninety-day readmission is the rehospitalization of a patient who underwent cervical surgery within 90 days of discharge due to unpredictable factors such as the same disease or complications [10, 11]. According to literature analysis, the readmission rate within 90 days after cervical surgery is 5–13% [9, 12]. However, potential risk factors for readmission are not analyzed in depth. Other studies on ninety-day readmission after cervical surgery are mostly focused on a single disease or surgical approach [13–15].

By summarizing the recently published studies on ninety-day readmission after cervical surgery, the purpose of our meta-analysis is to analyze the reasons for readmission and related risk factors, provide clinicians with certain literature references, and take timely countermeasures to reduce the risk of unplanned ninety-day readmission and lower operating costs for the healthcare system.

## Methods

### Search strategy

The first and third authors used computers to search databases such as Cochrane Library, PubMed, Web of Science, and Embase. The search time was set from the establishment of the database to August 1, 2022. We used the following search terms: cervical spine surgery; cervical surgery; 90-day readmission; ninety-day readmission; 90-day rehospitalization; ninety-day rehospitalization; and risk factors.

### Inclusion and exclusion principles

The inclusion principles: (1) research subjects: patients without ninety-day readmission were included in the control group and those who received ninety-day readmission after cervical surgery were included in the observation group; (2) research categories: randomized controlled trial (RCT) and retrospective cohort study (RCS) of risk factors for ninety-day readmission after cervical surgery; (3) evaluation indicators: age, American Society of Anesthesiology physical status (ASA) class, diabetes, hypertension, chronic heart diseases, chronic lung diseases, income, and payments for hospitalization; and (4) outcome: ninety-day readmission.

The exclusion principles: (1) research categories: editorial letter, animal studies, literature review, medical record report, republished literature, and conference paper; (2) literature content: incomplete data, unreproducible data, and statistical errors; and (3) access channels: not available.

### Statistics extraction and collection

Based on the inclusion and exclusion principles, the first and third authors retrieved 22,292 articles; they sorted out the documents with EndNote X9 software, checked the titles, and read abstracts and full texts carefully after ignoring the duplicates. The senior author could make the final decision in the case of disagreements. Finally, seven articles were included.

### Quality assessment

The second author independently assessed the seven included articles’ quality. In case of evaluation differences, the corresponding author hosted the discussion, and we resolved them together. RCSs were evaluated by NOS (Newcastle–Ottawa Scale). The evaluation indicators were as follows: case definition and representativeness, controls selection, controls definition, cases and controls comparability, exposure ascertainment, same methods of ascertainment for cases and controls, and non-response rate.

### Data analysis

We used Review Manager 5.4 software (https://www.cochrane.org/) provided by Cochrane for the meta-analysis. For continuous index data, mean difference (MD) was used as the effect index, and binary index data were used as the effect index. Risk ratio (RR) or odds ratio (OR) was used as effect indicators, respectively. A 95% confidence interval (CI) was also calculated. The value of *I*^2^ was used to judge the heterogeneity between studies: When *I*^2^ < 50%, the heterogeneity between the studies was small, and we used fixed effects model (FEM) for the analysis. When *I*^2^ > 50%, the heterogeneity between the studies was considerable, and subgroup analysis and sensitivity analysis were required to locate and clear the heterogeneity. When a *p* value < 0.05, the difference in the study was frequently considered statistically significant.

## Results

### Document screening

**Fig. 1:**
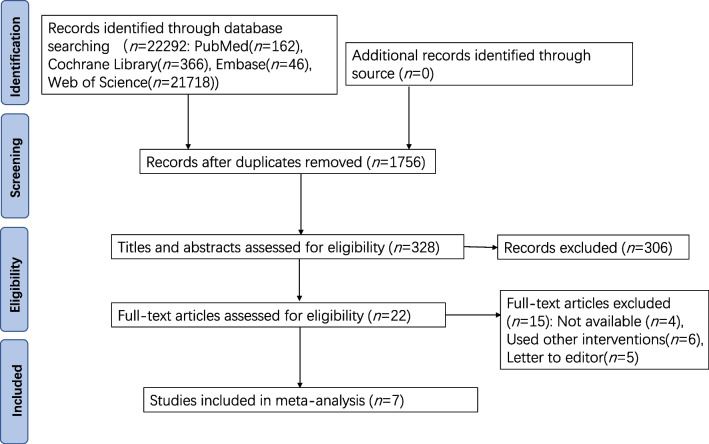
Flowchart of literature screening

Based on the established search strategy, 22,292 articles were collected. After the duplicate check, 1756 articles were retained. We meticulously implemented the inclusion and exclusion criteria after reviewing the titles and abstracts. Finally, seven articles were collected after thoroughly reading the full articles [9, 12–17]. The screening steps are displayed in Fig. 1.

### Basic characteristics of the included literature

This meta-analysis included seven studies with 222,490 patients. Of all the included studies, four articles provided age [12, 14, 16, 17], two articles provided ASA [9, 12], six articles provided diabetes [9, 12–15, 17], four articles provided hypertension [9, 13, 15, 17], three articles provided chronic heart diseases [9, 13, 15], five articles provided chronic lung diseases [9, 12, 13, 15, 17], three articles provided income [13, 15, 16], and four articles provided payments for hospitalization [13, 15–17]. The characteristics of the included literature are presented in Table 1.

**Table 1:** Basic characteristics of included studies

Author	Country	Study design	Procedure	Data source	Data collection Period	Patients	Outcomes
Rumalla2017	USA	RCS	PCF or PDWF	ND	2013.01–2013.09	29,990	③④⑤⑥⑦⑧
Rumalla2018	USA	RCS	ACDF or TDR	ND	2013.01–2013.09	72,688	③④⑤⑥⑦⑧
Dial2019	USA	RCS	ACDF	SC	2013–2017	1896	②③④⑤⑥
Goyal2019	USA	RCS	ACDF	ND	2012–2015	113,418	①⑦⑧
Elia2020	USA	RCS	OCF	ND	2016.01–2016.12	477	①③④⑥⑧
Schafer2020	USA	RCS	ACDF or PCF	SC	2014.02–2018.07	3762	①③
Badiee2021	USA	RCS	PCF	SC	2012–2020	259	①②③⑥

*USA* the United States; *RCS* retrospective cohort study; *ACDF* anterior cervical discectomy and fusion; *PCF* posterior cervical fusion; *PDWF* posterior decompression without fusion; *TDR* total disk replacement; *OCF* occipitocervical fusion; *ND* national database; *SC* single center; and ①Age, ②American Society of Anesthesiology physical status class, ③Diabetes, ④Hypertension, ⑤Chronic heart diseases, ⑥Chronic lung diseases, ⑦Income, ⑧Payments for hospitalization

### Evaluation of the quality of the studies

Seven RCSs were included into our analysis [9, 12–17]. The evaluation method turns to the NOS scores. The results of the evaluation are exhibited in Table 2.

**Table 2:** NOS scores

Study	Study design				Comparability	Exposure			Scores
	Case definition	Case representativeness	Selection of Controls	Definition of Controls	Comparability of cases and controls	Ascertainment of exposure	Same methods of ascertainment for cases and controls	Non-response rate	
Rumalla2017	★	★		★	★	★	★		6 stars
Rumalla2018	★	★	★	★	★	★	★	★	8 stars
Dial2019	★		★	★	★	★	★		6 stars
Goyal2019	★	★	★	★	★		★	★	7 stars
Elia2020	★		★	★	★	★	★	★	7 stars
Schafer2020	★	★	★	★	★	★	★	★	8 stars
Badiee2021	★	★		★	★	★	★	★	7 stars

Rated 6–8 stars as high-quality literature

### Meta-analysis

**Fig. 2:**
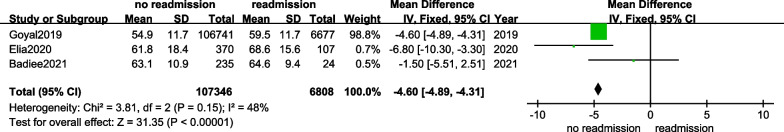
The forest plot of age

Four articles provided age data [12, 14, 16, 17]. Meta-analysis presented that the heterogeneity between included studies was high (*I*^2^ = 64%). Sensitivity analysis was conducted, and after one article was removed, the heterogeneity from the included studies was significantly diminished (*I*^2^ = 48%). FEM was used, and significant difference was found in the age between the readmission and no readmission groups (MD = − 4.60, 95%CI − 4.89–4.31, *p* < 0.00001), and the details are presented in Fig. 2.

**Fig. 3:**
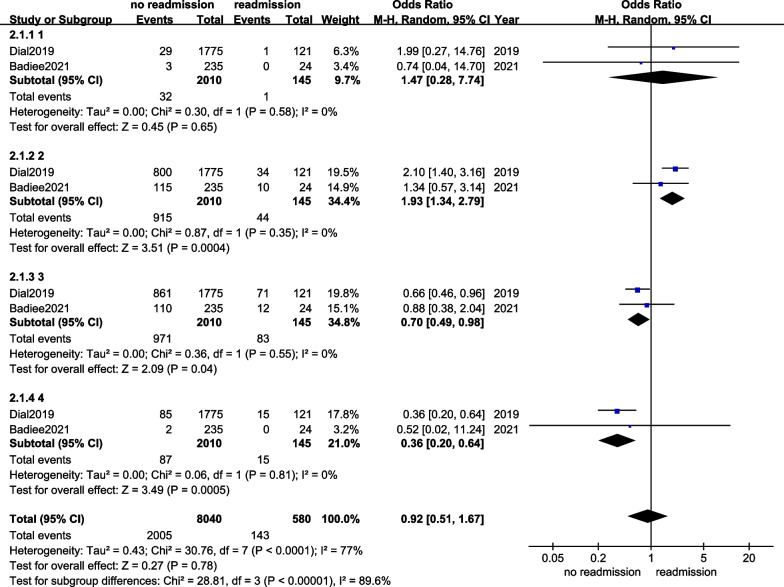
The forest plot of ASA

Two articles submitted ASA data [9, 12]. Subgroup analysis demonstrated that the heterogeneity between included studies was obvious (*I*^2^ = 77%). We used a random effects model (REM), and no difference was found in the ASA between the readmission and no readmission groups (OR = 0.92, 95%CI 0.51–1.67, *p* = 0.78), and the details are exhibited in Fig. 3.

**Fig. 4:**
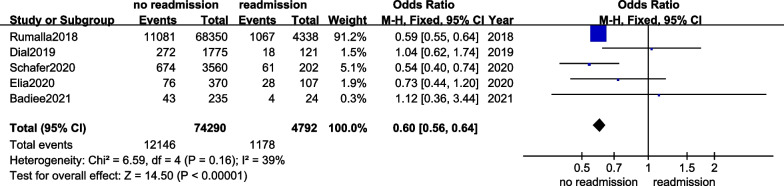
The forest plot of diabetes

Six articles offered diabetes data [9, 12–15, 17]. Meta-analysis presented that the heterogeneity between included studies was evident (*I*^2^ = 83%). We performed a sensitivity analysis. After one article was deleted, the heterogeneity was significantly lowered (*I*^2^ = 39%). FEM was used, and the results presented significant difference in the diabetes between the readmission and no readmission groups (OR = 0.60, 95%CI 0.56–0.64, *p* < 0.00001), see Fig. 4 for details.

**Fig. 5:**
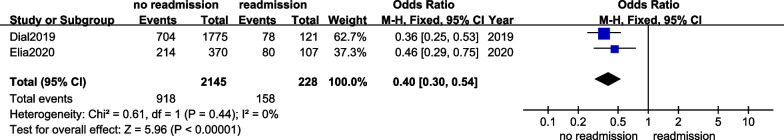
The forest plot of hypertension

Four articles provided hypertension data [9, 13, 15, 17]. Meta-analysis demonstrated that the heterogeneity between studies was obvious (*I*^2^ = 99%). Sensitivity analysis was performed, and after two articles were removed, the heterogeneity from the included studies was diminished (*I*^2^ = 0%). FEM was used, and significant difference was found in the hypertension between the no readmission and readmission groups (OR = 0.40, 95%CI 0.30–0.54, *p* < 0.00001), and the details are presented in Fig. 5.

**Fig. 6:**
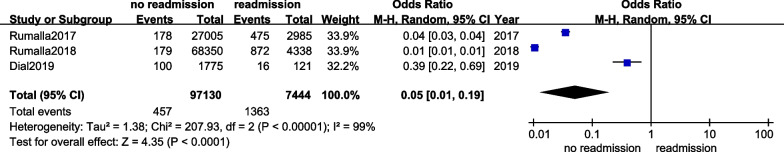
The forest plot of chronic heart diseases

Three articles provided chronic heart diseases data [9, 13, 15]. Meta-analysis indicated that the heterogeneity between studies was evident (*I*^2^ = 99%). We performed a sensitivity analysis. The heterogeneity between the two groups cannot be reduced or eliminated. We used a REM, and the results presented significant difference in the chronic heart diseases between the two groups (OR = 0.05, 95%CI 0.01–0.19, *p* < 0.00001), and the details are exhibited in Fig. 6.

**Fig. 7:**
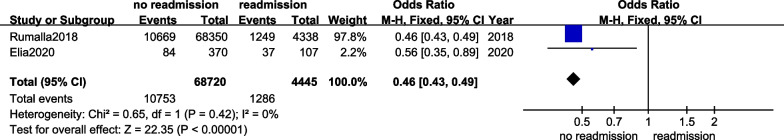
The forest plot of chronic lung diseases

Five articles submitted chronic lung diseases data [9, 12, 13, 15, 17]. Meta-analysis demonstrated that the heterogeneity between studies was high (*I*^2^ = 92%). Sensitivity analysis was performed, and after three articles were deleted, the heterogeneity between the groups can be significantly eliminated. We used a REM, and the results displayed significant difference in the chronic lung diseases between the groups (OR = 0.46, 95%CI 0.43–0.49, *p* < 0.00001), and the details are exhibited in Fig. 7.

**Fig. 8:**
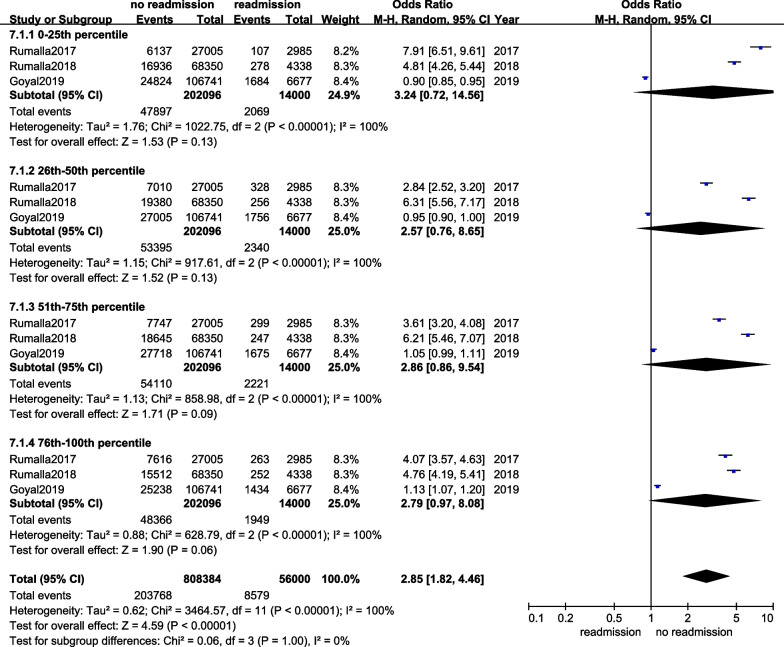
The forest plot of income

Three articles offered income data [13, 15, 16]. Subgroup analysis displayed that the heterogeneity between the studies was high (*I*^2^ = 100%). REM was used, and significant difference was found in the income between the two groups (OR = 2.85, 95%CI 1.82–4.46, *p* < 0.00001), and the details are shown in Fig. 8.

**Fig. 9:**
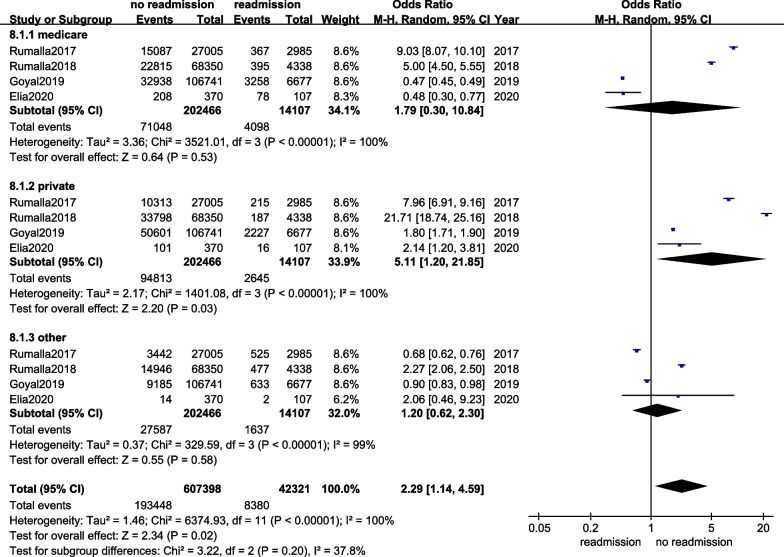
The forest plot of payments for hospitalization

Four articles provided payments for hospitalization data [13, 15–17]. Subgroup analysis displayed that the heterogeneity between included studies was obvious (*I*^2^ = 100%). We used a REM, and significant difference was found in the payments for hospitalization between the groups (OR = 2.29, 95%CI 1.14–4.59, *p* = 0.02), and the details are exhibited in Fig. 9.

## Discussion

Advances in surgical techniques have provided patients and physicians with more choices. With the increase in the number of cervical surgeries, people gradually pay attention to the quality of surgeries [18]. Physicians in the past have focused on surgical efficiency, morbidity, and mortality. The development of modern critical care medicine has greatly reduced postoperative mortality [19]. The incidence of postoperative complications is gradually being incorporated into the evaluation system of surgical treatment. Physicians’ observation of postoperative complications in patients is limited by the length of the patient’s hospital stay [17]. However, the promotion of the concept of fast recovery has shortened the average hospital stay of patients. Physicians have financial and promotion burdens that limit their ability to observe postoperative complications of patients and can only obtain relevant information through outpatient review or telephone follow-up. The quality of information obtained by doctors is affected by factors such as patients’ economic conditions, education level, and compliance [7, 20]. After the occurrence of postoperative complications, the patient needs to be readmitted for treatment, so the index of medical quality evaluation of readmission is derived [11]. Due to the special anatomical position, cervical surgery has the characteristics of high precision, high risk, and high cost. Once rehospitalization occurs within 90 days of cervical surgery, it will increase the patient’s physical pain and economic pressure and at the same time bring mental health burden and medical dispute risk to doctors [16, 21]. Therefore, it is of great significance to study the risk factors of ninety-day readmission to improve the quality of medical care.

Elderly patients undergoing cervical surgery may have preexistent injury to the balance of the cervical spine related to aging, stenosis of the intervertebral space, and cumulative damage. Compared to the younger patients, elderly population may have hidden diseases such as diabetes, lower immunity, higher postoperative infection rate, and longer recovery period [21, 22]. Our meta-analysis presented that the patient age in the no readmission group within ninety days after cervical surgery was younger than that in the ninety-day readmission group, and the difference was consistent with the previous conclusion [17].

Based on the patients’ organ status and hidden diseases, ASA classification system is used to evaluate the risk of anesthesia [23]. Multiple organ malfunction in elderly patients is a common issue, especially in patients aged over 80 years old, and those experiencing emergency surgery. In patients with higher ASA classification, these drugs used during anesthesia disturb the balance state of the patients’ physiology. When undergoing cervical surgery, the ability of human body to maintain physiological homeostasis is under tremendous pressure, and the physical difficulties faced by elderly patients are more arduous [24, 25]. Our analysis suggested that ASA class was not significantly correlated to the ninety-day readmission rate. However, in the subgroup analysis, the differences between the groups were statistically significant in the subgroups with ASA ratings 2, 3, and 4 (*P* = 0.0004, 0.04, and 0.005). It should be known that only two included studies reporting of ASA class. Consequently, our finding may be biased by the less samples and fewer studies.

Diabetes influences wound healing in patients undergoing cervical surgery. The underlying mechanism is as follows: Inflammatory response, reduced granulation tissue, and impaired angiogenesis at the wound location lead to slow wound healing [26]. Recent studies point out that in patients with diabetes, non-coding RNAs and epigenetic modifications are important causes that lead to abnormal transcription and influence activity of wound healing-related genes [27]. Ultimately, diabetes results in relatively poorer prognosis for patients. Our analysis found that the proportion of patients with ninety-day readmission with a history of diabetes was greater than that of the group without ninety-day readmission, and this group difference was significant.

Sympathetic nerve excitation caused by cervical degeneration or traumatic compression of vertebral artery is the pathological basis of high blood pressure [28]. Cervical degeneration increases vascular resistance. The continuous compression of the vertebral arteries leads to a continuous increase in blood pressure. These patients have hemodynamic disorders. When the decrease in blood flow rate is decompensated, the cytokines in the body are unbalanced. Vascular endothelial starts to dysfunction, releases inflammatory cytokines, and accelerates the transmission of painful feelings. Simultaneously, hypertension changes the structure and function of the body’s microvessels, causes microcirculation disorders, damages the target organs of hypertension, and increases the probability of cardiovascular and cerebrovascular complications after cervical surgery [29, 30]. Our findings found a higher incidence of hypertension in the ninety-day readmission group, which is consistent with previous study [3].

Patients with chronic heart diseases often take antiplatelet inhibitor such as aspirin as a secondary preventive treatment for myocardial infarction and ischemic cerebrovascular disease [31]. Cervical surgery, especially anterior surgery, requires strict intraoperative hemostasis. Once massive hemorrhage occurs, it will be difficult to stop the bleeding. The amount of bleeding will be large, and it will increase the difficulty of the operator’s operation. In severe cases, it will cause irreversible and catastrophic consequences. Epidural hematoma occurs after cervical surgery, and the cervical spinal cord will be rapidly damaged resulting in serious sequelae [32, 33]. In order to reduce intraoperative bleeding, patients are required to stop anticoagulant drugs so that patients are prone to postoperative cardiovascular and cerebrovascular complications and increase the risk of readmission [34, 35]. In this meta-analysis, patients who underwent ninety-day readmission after cervical surgery had a higher rate of chronic heart diseases, which is consistent with previous finding [9].

Chronic obstructive pulmonary disease (COPD) is one of the most common chronic lung diseases. Patients with COPD have systemic inflammatory response, long-term use of glucocorticoids, and lack of outdoor exercise. Extrapulmonary effects of COPD include osteoporosis and musculoskeletal dysfunction [36]. COPD patients have respiratory dysfunction, reduced lung volume, and abnormal thoracic activity, which complicate cervical surgery. With the aggravation of COPD, the risk of osteoporosis is higher [37]. COPD patients with osteoporosis may develop osteoporotic vertebral compression fractures after minor trauma or even daily coughing, which increases the probability of rehospitalization [38, 39]. Our analysis also showed that the probability of ninety-day readmission in patients with a history of chronic lung diseases was significantly higher than that in the group without COPD, and significant difference was found.

Patients with less income have heavier economic burden. The cost of cervical surgery and rehospitalization is a heavy economic expenditure. Conversely, patients with higher income levels have higher discretionary discretion and less financial pressure for readmission. Different payment methods also have a certain impact on the ninety-day readmission rate. Patients who use national medical insurance funds for settlement only need to pay lower medical expenses to obtain high-quality medical services, while patients using self-pay or commercial insurance are subject to certain restrictions when paying medical expenses [21, 40]. In our meta-subgroup analysis, we found statistically significant differences in income and payment patterns between the readmission group and the no readmission group within 90 days of cervical surgery.

Our study has the several limitations: All included papers written in English, and there was language bias; all included articles are RCSs, the general level of evidence was low; there was a deviation in research heterogeneity between the included studies; the signing for the consent document is a essential step for clinical research, but when it comes to the selection of treatment plans, there are interfering factors from human, which may lower the evidence level of the literature and ultimately influence the reliability of the conclusions of this study; and there are different rates of ninety-day readmission between the included studies, which may cause bias. Therefore, the conclusions of our meta-analysis still need to be further updated in the future with more samples, agencies, and studies with higher level of evidence.

## Conclusion

The ninety-day readmission rate after cervical spine surgery is 6.5%. Ninety-day readmission following cervical surgery is closely related to age, diabetes, hypertension, chronic heart diseases, chronic lung diseases, income, and payments for hospitalization.

## Data Availability

All the data used in our study are available on reasonable request.
